# A Mixture of Herbicides Dicamba and Glyphosate Causes Teratogenic Effects, Oxidative Stress, and Neurotoxicity in Zebrafish Embryos

**DOI:** 10.3390/toxics14050435

**Published:** 2026-05-14

**Authors:** Karoline Felisbino, Nathalia Kirsten, Shayane da Silva Milhorini, Karina Bernert, Rafaela Schiessl, Maiara Vicentini, Izonete Cristina Guiloski

**Affiliations:** 1 Faculdades Pequeno Príncipe, Av. Iguaçu, 333, Curitiba 80230-020, Brazil; karolinefel@hotmail.com (K.F.); nathalia.kirsten@aluno.fpp.edu.br (N.K.); shayanedasilva.s@gmail.com (S.d.S.M.); karina.bernert@aluno.fpp.edu.br (K.B.); rafaela.schiessl@aluno.fpp.edu.br (R.S.); 2 Instituto de Pesquisa Pelé Pequeno Príncipe, Av. Silva Jardim, 1632, Água Verde, Curitiba 80250-060, Brazil; 3 Department of Molecular and Cellular Biochemistry, College of Medicine, University of Kentucky, 741 S Limestone, Lexington, KY 40508, USA; 4 Instituto Carlos Chagas, Fundação Oswaldo Cruz, Rua Professor Algacyr Munhoz Mader, 3775, Curitiba 80350-010, Brazil; maiaravicentini@gmail.com

**Keywords:** acetylcholinesterase, antioxidant system, *Danio rerio*, pesticides, malformations

## Abstract

Exposure to pesticides during embryonic development can cause damage to health, especially when they are complex mixtures. This study evaluated the lethality, teratogenic effects, antioxidant system, and neurotoxicity in zebrafish (*Danio rerio*) embryos during a 96 h exposure to the herbicides dicamba (DIC) and glyphosate (GLY), alone and in a mixture. The embryos were exposed to 18 (D1) and 72 (D2) mg/L of DIC, 6 (G1) and 22 (G2) mg/L of GLY, and mixtures M1 (18 mg/L of DIC + 6 mg/L of GLY) and M2 (72 mg/L of DIC + 22 mg/L of GLY). The LC_50_ was 88.1 mg/L and 285.8 mg/L for GLY and DIC, respectively. M2 showed greater lethality at 48 and 72 h. The mixtures increased teratogenic effects. Generalized edema, predominant in embryos exposed to DIC, reached its maximum incidence between 48 and 72 h and then decreased. M1 delayed hatching by 72 h, while M2 accelerated it by 48 h. Yolk sac edema was significant in M1. Herbicides affected the antioxidant system differently: DIC reduced SOD activity, while GLY increased it. Additionally, DIC and M2 increased acetylcholinesterase activity, indicating neurotoxicity. This is the first study to report the effects of these herbicide mixtures on zebrafish embryos, highlighting the complexity and severity of their impact on embryonic development. Therefore, the importance of the controlled use of these pesticides is emphasized to avoid harmful effects on non-target organisms.

## 1. Introduction

Exposure to various xenobiotics from food, water, and air [1,2], can cause irreversible changes in the structure and function of living organisms, including effects on genetic material and possible contributions to cancer [3,4,5]. In addition, it leads to an increase in the production of reactive oxygen species (ROS) in cells and, consequently, to oxidative stress [6,7]. Among the xenobiotics reported to cause a loss of redox homeostasis are pesticides [8].

Brazil is among the world’s leading consumers of pesticides, underscoring the need for strict monitoring. The regulatory status of these substances varies across regions, where they may be restricted, banned, or subject to specific limitations. For example, the herbicide dicamba (DIC) is banned in India, glyphosate (GLY) is banned in parts of Asia, and atrazine is banned in the European Union; however, these pesticides remain permitted in Brazil. This situation raises serious concerns regarding potential impacts on human health and the environment [9,10,11]. Notably, the use of herbicides has overtaken insecticides and fungicides since 2010 [12].

Combinations of various chemical substances, each with specific effects on different organisms, are commonly used in agricultural areas and have the potential to be transported by water bodies, posing risks to non-target species [13]. Although the use of DIC and GLY is widely documented in agriculture, there is a lack of studies investigating the effects of these compounds, both isolated and in combination, on aquatic organisms, particularly zebrafish. One of the aims of this study is to fill this gap in the literature by assessing the impacts of DIC and GLY on the development, neurotoxicity, and antioxidant system alterations during zebrafish embryo development.

For more than five decades, DIC has been used to control weeds and is generally used in combination with GLY. This mixture is due to the increasing number of plants that have developed resistance to GLY, making DIC a more viable option [14,15,16]. Studies indicate that mixing these herbicides can alter their chemical properties [17,18] and their effects in terms of toxicity and efficiency [15,19]. Thus, understanding the combined effects of these herbicides on non-target organisms is crucial for evaluating their potential risks to ecosystems and human health.

The zebrafish, *Danio rerio*, is an efficient model in human disease research [20] and contaminant assessment [21]. It is approximately four centimeters long, has low maintenance costs, has high fecundity, and matures quickly [22,23]. Its external reproduction facilitates the collection of translucent eggs, ideal for teratogenic studies [24]. Zebrafish have about 70% genomic similarity to humans, and genetic sequencing has enabled genotoxicological studies and various biomarkers, consolidating it as a good model in biological research [21].

Assessing the effects during embryo development is crucial to understanding the impact of these substances, especially in early life stages, when organisms are most susceptible to environmental stressors. This study evaluates whether DIC in mixture with GLY causes effects on development, neurotoxicity, and antioxidant system alterations and compares these effects with those observed when the herbicides are tested individually. We hypothesize that the mixture of DIC and GLY induces greater toxic effects than individual exposures.

## 2. Materials and Methods

### 2.1. Herbicides

The herbicide glyphosate (GLY—CAS 1071-83-6, Zapp Pro^®^ from Syngenta, São Paulo, Brazil; 620 g/L) had its standard solution prepared at a concentration of 70,000 mg/L. Dicamba (DIC—CAS 1918-00-9, Atectra^®^ from BASF SA, São Paulo, Brazil; 480 g/L) had its standard solution prepared at a concentration of 18,000 mg/L. The dilutions, in maintenance water, for the tests were subsequently made from the standard solutions of each herbicide.

### 2.2. Fish Keeping and Egg Collection

For reproduction, wild-type zebrafish (*Danio rerio*) were maintained in 26 L aquariums containing 20 fish each, with an approximate 1:1 male-to-female ratio, under controlled conditions (pH 7.2 ± 0.25, temperature 26 ± 2 °C, and a 14:10 h light/dark photoperiod). The fish were fed three times daily with Alpacon^®^ commercial feed (minimum crude protein 450 g/kg; 45%). Water quality parameters were routinely monitored every 2 days, including temperature, pH, and dissolved oxygen. Ammonia concentrations ranged from 0 to 1.5 ppm, with most measurements remaining below 0.5 ppm, while nitrite levels varied between 0 and 2.8 ppm, typically within 0.25–1.0 ppm. Aquariums were cleaned daily to remove excess solid waste, such as feces and leftover feed, ensuring stable rearing conditions.

For spawning, adults were kept together overnight in their home tanks, and a basket breeding insert was placed inside the aquarium in the evening. Eggs were collected the following morning, approximately 1 h after lights-on, when the breeding insert was removed to separate the adults from the embryos and prevent egg predation.

The test took place before the embryos reached 16 cells, and lasted 96 h, with only viable fertilized eggs being transferred to 24-well plates, according to OECD 236 [25]. Fertilized eggs were selected using a stereomicroscope (magnification ≥ 30×), choosing those in the process of cleavage and without visible irregularities (e.g., asymmetry or vesicle formation) or lesions on the chorion. The plates containing the embryos were kept in a temperature-controlled incubator (Biological Oxygen Demand incubator, B.O.D.—SSGF, SolidSteel—Piracicaba, SP, Brasil) at 28 °C, under a 14:10 h light/dark photoperiod to mimic the natural circadian cycle.

### 2.3. Median Lethal Concentration (LC_50_)

The initial concentrations selected for the herbicides were 70 mg/L for glyphosate (GLY) and 18 mg/L for dicamba (DIC), based on the Drinking Water Equivalent Level (DWEL) [26]. For DIC, concentration selection was further supported by previously reported data from our group [27], in which zebrafish embryos were exposed to 4.5, 18, 72, and 288 mg/L, resulting in an LC50 of approximately 285 mg/L after 96 h of exposure. These values were used to guide the experimental design in the present study.

For GLY, concentrations of 6, 14, 32, 70, and 154 mg/L were tested to determine the LC50 under the same experimental conditions. These concentrations were selected based on the DWEL value (70 mg/L), which was used as a central reference point, with additional concentrations distributed below and above this value to allow the evaluation of both sublethal and lethal effects and to enable accurate LC50 determination.

Embryos were individually placed in 24-well plates (one embryo per well), with one plate assigned per concentration per replicate. Each experimental condition included three independent biological replicates (*n* = 20 embryos per replicate; total *n* = 60 embryos per group). All treatments were conducted alongside a negative control (system water) and a positive control (3,4-dichloroalanine, 4 mg/L).

Lethality was assessed every 24 h over a 96 h exposure period, based on the following endpoints defined by OECD guideline 236 [25]: egg coagulation, absence of somite formation, non-detachment of the tail, and absence of heartbeat.

### 2.4. Teratogenicity and Hatching Rate Test

After defining the LC_50_ of the herbicides, teratogenicity and hatching rate tests were carried out. The following groups were used to assess teratogenic effects, hatching rate, and biochemical assays: control (C); dicamba 1 (D1: 18 mg/L); dicamba 2 (D2: 72 mg/L); glyphosate 1 (G1: 6 mg/L); glyphosate 2 (G2: 22 mg/L); mixture 1 (M1: D1 + G1); and mixture 2 (M2: D2 + G2). The teratogenicity test was conducted in 24-well plates under the same conditions as the LC_50_ assay, and all experiments were performed in triplicate. Each experimental group consisted of 20 embryos per replicate, with one embryo placed per well in 24-well plates. All experiments were performed in triplicate (three independent biological replicates), resulting in a total of 60 embryos per group.

Malformations and hatching frequency were analyzed every 24 h for 96 h using a stereomicroscope (≥30× magnification). Ocular malformations were identified by asymmetry or reduction in eye size, while tail and spine malformations were characterized by curvatures, kinking, or shortening of the posterior axis. Pericardial edema was identified by the presence of fluid accumulation in the cardiac region, and dwarfism by a noticeable reduction in total body length compared with age-matched controls. Yolk malabsorption was recognized by the persistence of a large, grayish or opaque yolk sac that did not undergo normal resorption over time, indicating impaired nutrient utilization. Additional alterations screened included yolk sac edema, absence of otoliths, poor pigmentation, bleeding, spasms, malformed fins, and absence of head formation.

### 2.5. Exposure to Herbicides

The zebrafish embryos were exposed to the herbicides in 24-well plates. Each embryo was individually placed in a single well (one embryo per well), totaling 20 embryos per experimental group. In addition, four wells in each plate were used as internal controls, containing embryos maintained in control water without herbicide exposure. A separate control group was also included, following the same experimental design, with embryos exposed only to maintenance water.

Some specific characteristics were observed to assess the lethality effects, including the coagulation of the eggs, which could occur after 24 h, resulting in eggs with a milky white color that turned dark when observed under a stereomicroscope. In addition, we recorded the absence of somite formation after 24 h, indicated by the lack of spontaneous movements, and the failure of the tail to separate from the yolk sac after 24 h until the last embryo elongated. The absence of a heartbeat was considered an indicator of lethality and was recorded when no heartbeats were observed over a continuous 60 s observation period, starting from 48 h onwards, under 80× magnification.

In addition to the lethality parameters, teratogenicity parameters were observed for each embryo every 24 h. Among the defects observed were cardiac edema, yolk sac edema, otolith absence, generalized edema, dwarfism, pigmentation, hemorrhage, spine and tail malformation, and developmental delay. The tests were conducted with a control group.

### 2.6. Evaluation of Antioxidant System and Acetylcholinesterase Activity

Biochemical biomarkers were analyzed using pools of 10 larvae per experimental replicate (*n* = 10 per group). Biochemical biomarkers were analyzed using pools of 10 larvae per replicate. Each experimental group consisted of 10 independent pools (*n* = 10), resulting in a total of 100 larvae per group. The samples were homogenized in 0.1 M phosphate buffer, pH 7.0, using a micro-homogenizer Ultra Turrax^®T^T10, IKA, Staufen, Germany. The homogenate was centrifuged at 12,000× *g* for 15 min at 4 °C, according to Felisbino et al. [27]. The supernatants were stored at −80 °C until analysis. The supernatants were used to assess superoxide dismutase (SOD), glutathione peroxidase (GPx), and acetylcholinesterase (AChE) activities, and total protein concentration.

To assess SOD activity, the Gao et al. [28] method, reliant on SOD’s capability to avoid pyrogallol auto-oxidation, was employed. In microtubes, 40 µL of the sample was added to 885 µL of 1 M Tris/5 mM EDTA buffer (pH 8.0) and 50 µL of pyrogallol (15 mM). The solution was protected from light and incubated for 30 min. To stop the reaction, 25 µL of 1 N HCl was added. Subsequently, 200 µL of the solution were transferred to a microplate. Measurements were taken at 440 nm and the results were reported in SOD units per milligram of protein.

For GPx activity evaluation, the method developed by Paglia and Valentine [29] was used. Samples (10 μL) were mixed with 130 μL of solution 1 (0.1 M sodium phosphate buffer—pH 7.0, sodium azide at 3.1 mM, NADPH at 0.31 mM, GSH at 3.08 mM, and glutathione reductase at 1.54 U/mL). After two-minutes, 60 μL of solution 2 (hydrogen peroxide—H_2_O_2_ at 5 mM and 0.1 M sodium phosphate, pH 7.0) was added. Readings were taken at 340 nm at 30 s intervals for 5 min and the GPx activity was quantified as nmol·min^−1^·mg^−1^ protein.

AChE activity in larvae was measured using the method of Ellman et al. [30]. The reading was carried out in a spectrophotometer at 405 nm every 30 s for 5 min and the enzyme activity was expressed as nmol·min^−1^·mg^−1^ protein.

The total protein concentration was determined using Bradford [31], with bovine serum albumin as a standard. In a microplate, 10 µL of the sample was combined with 250 µL of Bradford’s reagent (Sigma-Aldrich^®^—Darmstadt, Germany) and absorbance measurements were made at 595 nm. The results were reported in milligrams of protein.

### 2.7. Statistical Analysis

For the LC_50_ analysis, the data were tabulated, log-transformed, and normalized. Non-linear regression statistics, specifically dose–response analysis, were carried out using the Prism^®^ 5 software. The Kolmogorov–Smirnov test was used to assess the normality of the data. The biochemical and teratogenicity analyses were carried out using analysis of variance (ANOVA) with a significance level of *p* < 0.05, together with Tukey’s post-test to make comparisons between the means. For the biochemical analyses, a number of 10 individuals per group was used, while for the teratogenicity analyses, each group consisted of 20 individuals (carried out in triplicate). The results are expressed as mean ± standard error of the mean (SEM).

To analyze the data in an integrated way, the Integrated Biomarker Response Index (IBRv2) was calculated according to the method described by Beliaeff and Burgeot [32], as modified by Sanchez et al. [33]. Briefly, biomarker responses were standardized by calculating the deviation of each value relative to the control group mean, followed by logarithmic transformation to reduce variance and normalize the data distribution. For each biomarker, the standardized values were adjusted according to the direction of the biological response (activation or inhibition), and a scoring system was applied. The resulting scores were then summed to obtain the IBRv2 index for each experimental group, representing the overall biological stress level. Radar (star) plots were used to visually represent the contribution of each biomarker to the integrated response.

## 3. Results

### 3.1. Median Lethal Concentration (LC_50_) of Herbicides

**Figure 1:**
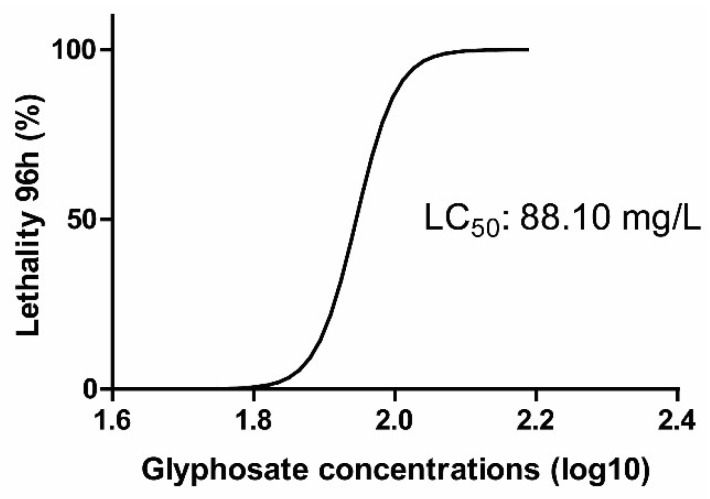
Concentrations–response curve of glyphosate herbicide exposure for lethality with data in percentage and concentrations expressed in log; 95% confidence interval; R^2^ = 0.3626 HILLSLOPE = 15.21.

The LC50 of dicamba (DIC) was 285.8 mg/L, as previously reported [27]. For glyphosate (GLY), the estimated LC50 was 88.1 mg/L (Figure 1). However, the dose–response model for GLY showed a low goodness of fit (R^2^ = 0.3626) and high variability in parameter estimates, indicating that this value should be interpreted with caution (Figure 1).

### 3.2. Lethality, Teratogenicity and Hatching Rate Test

**Figure 2:**
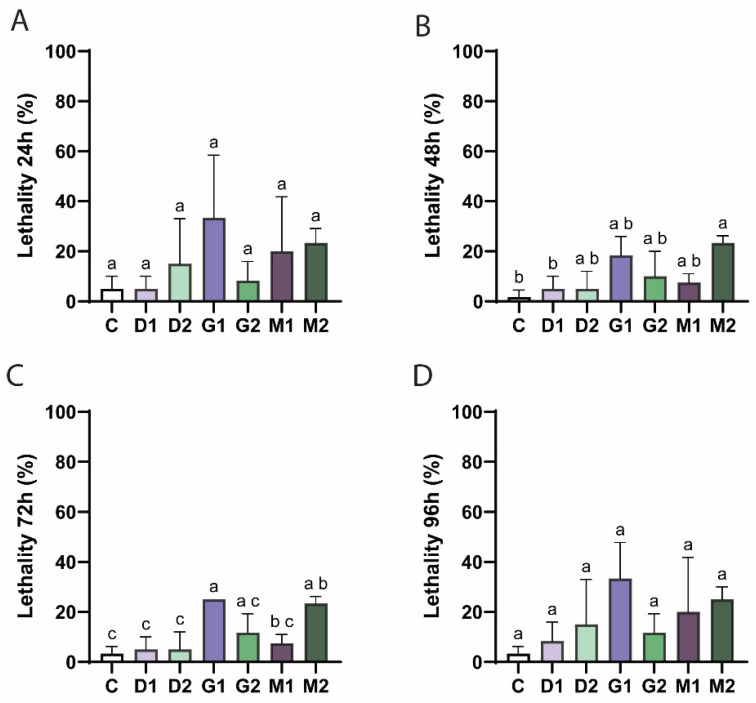
Lethality of zebrafish embryos after 24 (**A**), 48 (**B**), 72 (**C**), and 96 (**D**) hours of exposure to herbicides. C: Control; dicamba 1 (D1): 18 mg/L; dicamba 2 (D2): 72 mg/L; glyphosate 1 (G1): 6 mg/L; glyphosate 2 (G2): 22 mg/L; mixture 1 (M1): D1 + G1; mixture 2 (M2): D2 + G2. Data are expressed as percentage of lethality (mean ± SEM). Different letters indicate statistically significant differences among groups (*p* < 0.05; one-way ANOVA followed by Tukey’s test). Tests carried out in triplicate with *n* = 20 for each group.

Embryonic lethality (Figure 2A) was observed from the first 24 h of exposure. Control mortality remained below 10% throughout the experiment, meeting the validity criteria established by OECD TG 236 [25]. Although the G1 group exhibited a higher mean lethality at 24 h compared to the other groups, no statistically significant differences were detected among treatments at this time point (*p* > 0.05).

After 48 h (Figure 2B), significant differences among groups were observed (*p* < 0.05). The M2 group showed higher lethality compared to groups with different statistical lettering, while sharing similarities with intermediate groups, indicating partial overlap in responses.

At 72 h (Figure 2C), significant differences were also observed (*p* < 0.05), with the G1 group presenting higher lethality compared to the control (C) and D1 groups. The M2 group showed intermediate responses, sharing statistical similarity with both higher and lower response groups.

After 96 h (Figure 2D), although differences in mean lethality were observed, no statistically significant differences were detected among groups (*p* > 0.05).

**Figure 3:**
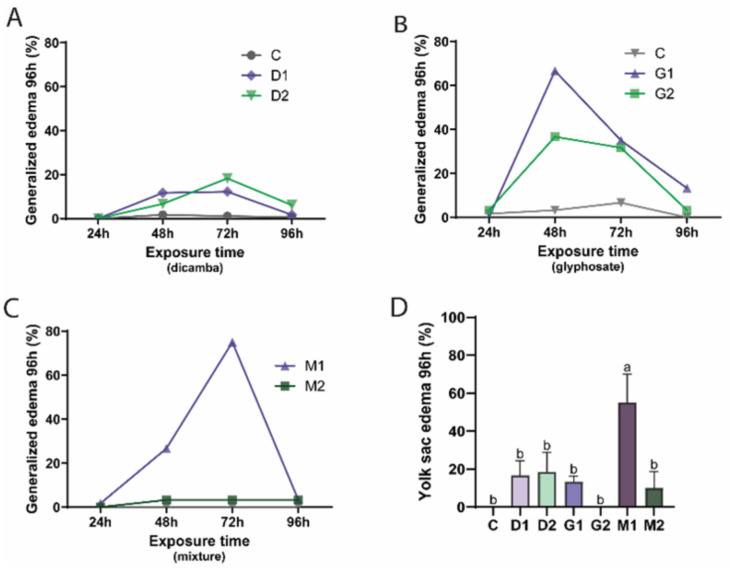
Frequency of teratogenic effects in zebrafish embryos after 24, 48, 72, and 96 h of exposure to herbicides. (**A**) Dicamba (D1: 18 mg/L; D2: 72 mg/L); (**B**) glyphosate (G1: 6 mg/L; G2: 22 mg/L); (**C**) mixtures (M1: D1 + G1; M2: D2 + G2); (**D**) yolk sac edema after 96 h of exposure. Data are expressed as the percentage of affected embryos (%) (mean ± SEM). Different letters indicate statistically significant differences among groups (*p* < 0.05; one-way ANOVA followed by Tukey’s test). Experiments were performed in triplicate with *n* = 20 embryos per replicate (total *n* = 60 per group). C: Control.

The results indicated the occurrence of generalized edema, characterized by fluid accumulation in the yolk sac and cardiac regions, which in some cases extended to other areas of the body. This effect showed an increase in the first hours of exposure, normalizing after 72 h. In the groups exposed to the herbicide DIC (D1 and D2), there was a greater number of embryos with edema compared to the control group. In addition, the D2 group (72 mg/L) showed a greater number of individuals with generalized edema, peaking at 72 h (Figure 3A). For the herbicide GLY, although both groups, G1 and G2 (6 and 22 mg/L; respectively), caused a greater number of edemas compared to the control group, the embryos developed more edemas when exposed to the lower concentration (G1), with the peak occurring after 48 h of exposure (Figure 3B). Concerning the mixtures, M1 showed similar behavior to D2, with the peak also occurring after 72 h of exposure and decreasing thereafter. However, M2 was similar to the control, showing fewer adverse effects (Figure 3C).

Both herbicides caused yolk sac edema (Figure 3D), which was one of the most commonly observed effects. At 96 h of exposure, M1, a mixture of the two lowest concentrations of the herbicides, caused a significant increase (*p* = 0.0004; F_(6,14)_ = 9.086) in yolk sac edema both compared to the control and the other groups, including D1, D2, G1, G2, and M2.

**Figure 4:**
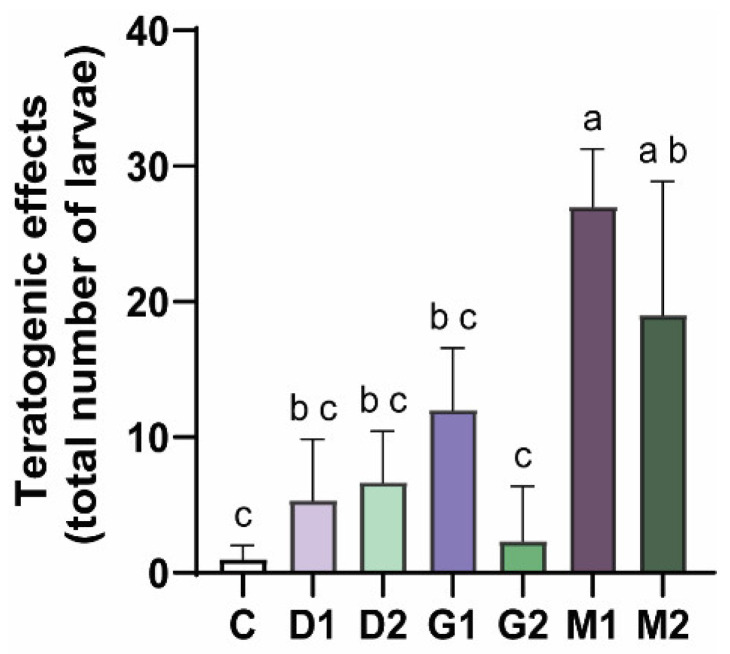
Total number of teratogenic events observed in zebrafish embryos after 96 h of exposure to herbicides. C: Control; dicamba 1 (D1): 18 mg/L; dicamba 2 (D2): 72 mg/L; glyphosate 1 (G1): 6 mg/L; glyphosate 2 (G2): 22 mg/L; mixture 1 (M1): D1 + G1; mixture 2 (M2): D2 + G2. Data represent the total number of teratogenic events (mean ± SEM), with multiple effects possibly occurring in the same individual. Different letters indicate statistically significant differences among groups (*p* < 0.05; one-way ANOVA followed by Tukey’s test). Experiments were performed in triplicate with *n* = 20 embryos per replicate.

Regarding the total number of teratogenic events observed, a significant overall effect was detected among groups (one-way ANOVA, *p* = 0.0008; F_(6,12)_ = 9.631). Post hoc analysis (Tukey’s test) revealed that the mixture groups showed increased numbers of teratogenic events compared to the control group. M1 presented a significantly higher number of events compared to all individual herbicide treatments (D1, D2, G1, and G2). The M2 group also showed an increased number of teratogenic events compared to the control and G2 groups (Figure 4).

**Figure 5:**
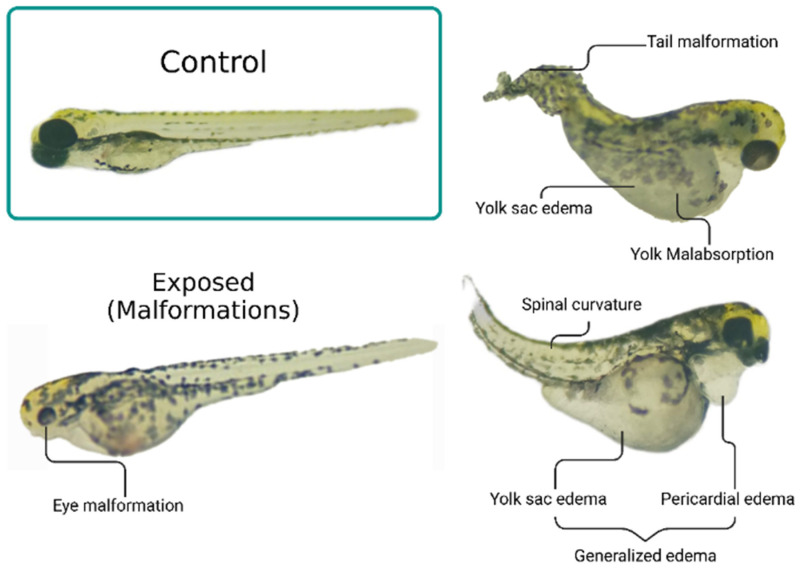
Representative morphological alterations observed in zebrafish larvae after herbicide exposure. Control larvae show normal development, while exposed larvae present abnormalities including eye malformation, spinal curvature, yolk sac edema, pericardial edema, yolk malabsorption, and tail malformation. Generalized edema is characterized by the simultaneous presence of yolk sac and pericardial edema.

As for the types of malformations and effects found, in addition to yolk sac and generalized edema, the larvae also had cardiac edema, yolk malabsorption, dwarfism, ocular malformation, and malformation of the spine and tail (Table 1). After yolk sac edema, yolk malabsorption was the second most prevalent, with all groups (including control) presenting it, but only D1 and M2 had a significant increase compared to C (*p* < 0.0001; F_(6.14)_ = 12.79). Representative images illustrating the main developmental abnormalities observed in exposed groups are presented in Figure 5.

**Figure 6:**
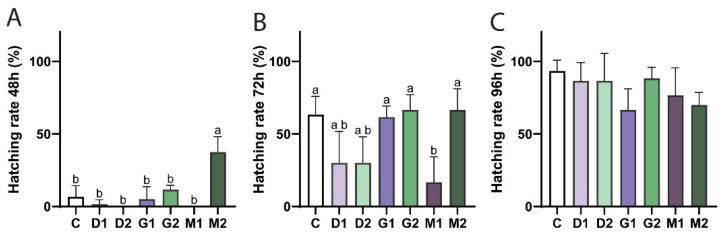
Hatching of zebrafish embryos after 48, 72, and 96 h of exposure to herbicides. C: Control (maintenance water); dicamba 1 (D1): 18 mg/L; dicamba 2 (D2): 72 mg/L; glyphosate 1 (G1): 6 mg/L; glyphosate 2 (G2): 22 mg/L; mixture 1 (M1): D1 + G1; mixture (M2): D2 + G2. Tests carried out in triplicate with *n* = 20 for each group. Data in percentage. Different letters indicate statistically significant differences between the groups (*p* < 0.05) after ANOVA followed by Tukey’s multiple comparison test (mean ± SEM).

During the first 48 h of exposure, some embryos hatched (Figure 6A). M2 showed a significant increase in hatching compared to the control and to the other groups (D1, D2, G1, G2, and M1) (*p* = 0.0006; F_(6,13)_ = 8.770). After 72 h of exposure (Figure 6B), the number of hatched embryos increased in all groups, including the control, compared to 48 h. Only the M1 group showed a significant reduction in hatching compared to the control at 72 h. At 96 h, no significant differences in hatching were observed among the groups (Figure 6C).

### 3.3. Antioxidant System and Acetylcholinesterase Activity

**Figure 7:**
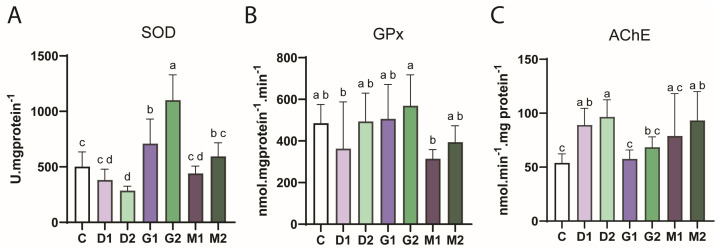
Biochemical markers in zebrafish embryos after 96 h of exposure to herbicides. C: Control (maintenance water); dicamba 1 (D1): 18 mg/L; dicamba 2 (D2): 72 mg/L; glyphosate 1 (G1): 6 mg/L; glyphosate 2 (G2): 22 mg/L; mixture 1 (M1): D1 + G1; mixture 2 (M2): D2 + G2). A: SOD = superoxide dismutase activity; B: GPx = glutathione peroxidase activity; C: AChE = acetylcholinesterase activity. Different letters indicate statistically significant differences between the groups (*p* < 0.05), after ANOVA followed by Tukey’s multiple comparison test (mean ± SEM). *n* = 10.

Evaluation of the antioxidant system showed that the highest concentration of dicamba (72 mg/L; D2) reduced SOD activity (*p* < 0.0001; F_(6.58)_ = 32.19). In contrast, exposure to the herbicide GLY caused a significant increase in SOD activity in both groups, G1 and G2 (6 and 22 mg/L, respectively). M1 and M2 groups did not differ from the control group but were different from each other. M1 also showed a statistical decrease in relation to the G1 and G2. However, M2 showed a significant decrease compared to G2 and a significant increase compared to D2 (Figure 7A).

GPx activity (Figure 7B) did not differ significantly from control in groups exposed to herbicides (D1, D2, G1, G2, M1, and M2). However, the G2 group showed a significant difference (*p* = 0.007; F_(6.55)_ = 3.380) to the D1 and M1 groups.

Neurotoxicity was assessed by analyzing the activity of the enzyme AChE, which showed increased activity in embryos exposed to the two concentrations of the herbicide DIC (D1 and D2 groups), and for exposure to M2, when compared to the control (*p* < 0.0001; F_(6,60)_ = 7.236). The DIC groups (D1 and D2) also showed a significant increase in AChE activity when compared to the groups exposed to GLY (G1 and G2) and M1 (Figure 7C).

### 3.4. Integrated Biomarker Response Index (IBRv2)

The Integrated Biomarker Response Index (IBRv2) was used as a tool to summarize multiple biomarker responses into a single value, allowing the comparison of overall stress levels among treatments. This index integrates biochemical (SOD, GPx, AChE) and morphological endpoints (teratogenic effects) by standardizing and combining their responses, providing a comprehensive overview of organismal stress. However, it is important to note that IBRv2 represents an integrated response and does not provide direct mechanistic insight into specific pathways.

These results indicate that the effects of the herbicide mixtures depend on concentration and are not consistently associated with increased stress compared to individual compounds. Although certain mixtures exhibited higher integrated biomarker responses, this pattern was not consistent across all conditions, indicating that interactions between herbicides may result in variable, non-linear toxicological outcomes rather than a uniform increase in stress.

**Figure 8:**
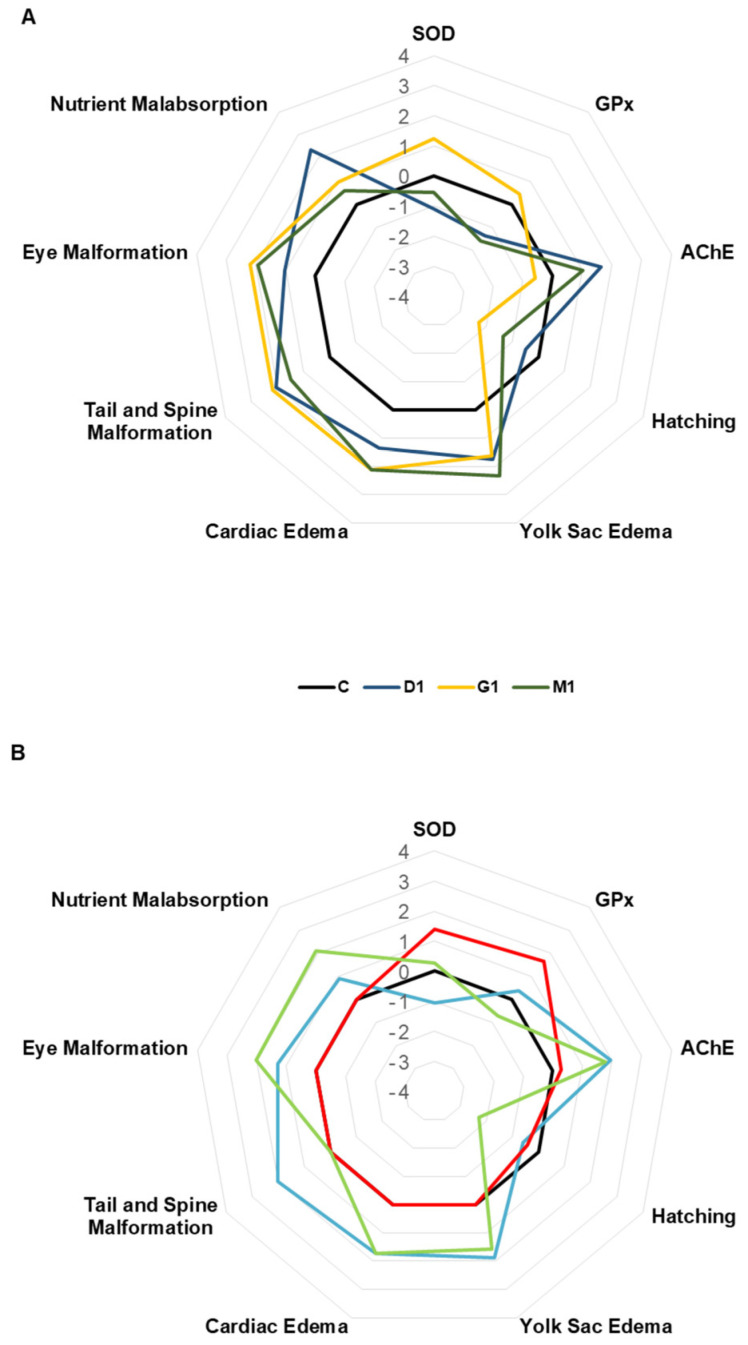
Integrated Biomarker Response (IBRv2) profiles after 96 h of exposure to herbicides. Radar plots represent the standardized responses of biochemical (SOD, GPx, AChE) and morphological endpoints (teratogenic effects, hatching, edema, malformations, and nutrient malabsorption) used to calculate the IBRv2 index. C: Control (system water); dicamba 1 (D1): 18 mg/L; dicamba 2 (D2): 72 mg/L; glyphosate 1 (G1): 6 mg/L; glyphosate 2 (G2): 22 mg/L; mixture 1 (M1): D1 + G1; mixture 2 (M2): D2 + G2.

The isolated concentrations of dicamba (18 mg/L; IBRv2: 13.13) and glyphosate (6 mg/L; IBRv2: 13.62) showed similar IBRv2 values compared to their corresponding mixture (IBRv2: 13.01; Figure 8A). In contrast, at higher concentrations, the mixture (IBRv2: 12.47) exhibited a higher integrated response than dicamba (72 mg/L; IBRv2: 11.72) and glyphosate (22 mg/L; IBRv2: 3.76) when tested individually (Figure 8B).

## 4. Discussion

The lower estimated LC50 of glyphosate compared to dicamba suggests a higher sensitivity of zebrafish embryos to glyphosate at lower concentrations. However, this interpretation should be made with caution, as the dose–response model for glyphosate showed a low goodness of fit, indicating variability in the data. In general, reported LC50 values for glyphosate in zebrafish embryos vary widely depending on experimental conditions, particularly formulation and exposure parameters. For example, studies summarized in the literature report LC50 values around 66.04 mg/L at 48 hpf, with increased mortality observed at concentrations above 50 mg/L and complete lethality at higher doses [34]. In agreement with this variability, Tóth et al. [35] demonstrated that the LC50 of glyphosate differs significantly among commercial formulations, with values ranging from 30.8 mg/L (Roundup^®^ Mega) to 160.0 mg/L for analytical grade glyphosate, highlighting the influence of co-formulants on toxicity.

In addition, Ruiz de Arcaute et al. [19] reported that the toxicity of glyphosate–dicamba mixtures depends on the proportion of each herbicide, with interactions ranging from additive to slightly antagonistic. This supports the results observed in the present study, in which the toxic effects varied according to the concentration and combination of the herbicides, rather than following a consistent pattern of increased toxicity in mixtures.

In the beginning of the lethality assay the impact of the herbicides on the survival of the embryos was seen at 24 h of exposure as the M2 group showed a high lethality, when compared to the control. The effect continued to occur at 48 h of exposure, when M2 showed a significant increase in embryo mortality compared to the control group. This increase in lethality suggests that the combination of these herbicides, at higher concentrations, began to have a detrimental effect on the embryos. G1 also recorded an increase at 48 h of exposure, but it did not yet reach statistical significance in relation to the control.

In our work, we selected concentrations based on the Drinking Water Equivalent Level (DWEL), which were 70 mg/L for the herbicide glyphosate and 18 mg/L for dicamba [26]. Surprisingly, our findings revealed that even at or below concentrations considered safe according to regulations, these herbicides induced adverse developmental effects, including lethality. Conversely, a separate study simulated prolonged mammal exposure to low, supposedly safe doses of pesticides, employing Wistar rats as an experimental model. The authors demonstrated that GLY led to detrimental effects such as apoptosis. Moreover, when combined with DIC and 2,4-D, the impact intensified significantly, hinting at an additive influence of the herbicide mixture. In this study, females were exposed to GLY at regulatory doses deemed safe (ADI—Acceptable Daily Intake; 0.5 mg/kg body weight/day—and NOAEL—No Observed Adverse Effect Level; 50 mg/kg body weight/day) during gestation, along with a combination of herbicides containing GLY, DIC, and 2,4-D. The outcomes observed included renal cell dysfunction in mothers and antiandrogenic effects in their offspring [36].

At 72 h, the G1 and M2 groups showed a significantly higher mortality rate compared to the control group. These results show that GLY, even at relatively low concentrations, and the combination of D2 and G2 had a negative impact on embryos after 72 h of exposure, as in the study by Ruiz de Arcaute et al. [19], which showed that GLY was more lethal than DIC.

In addition, the M2 group showed accelerated hatching as early as after 48 h of exposure, unlike all other groups. Although this finding may initially appear contradictory to the increased lethality observed in the same group, accelerated hatching may represent a stress-related response to adverse environmental conditions, as early developmental processes are highly sensitive to environmental stressors [37]. The presence or absence of the chorion can influence developmental processes, behavior, and gene expression in zebrafish embryos exposed to chemicals. Furthermore, chorion loss has been associated with increased embryo vulnerability and toxicity [38], which may contribute to the higher lethality observed in this group. In addition, D1 and M2 showed increased yolk malabsorption at 96 h of exposure. Impaired nutrient absorption is directly linked to developmental disturbances and may also affect oxygen uptake and transport across membranes [39], further contributing to reduced survival.

Unlike M2, the M1 group, which received the mixture with the lowest concentrations, showed a delay in embryo hatching over 72 h. In addition, during this same period, there was an increase in the generalized edema effect. Subsequently, a greater number of embryos with yolk sac edema and total teratogenic effects were observed during the 96 h exposure period. These observations indicate an alteration in embryonic development, possibly due to the accentuated inflammatory process, since inflammatory mediators are related to delayed embryonic development [40].

These findings are consistent with those reported by Liu et al. [41], who demonstrated that glyphosate exposure in zebrafish embryos induces developmental alterations such as premature hatching, pericardial and yolk sac edema, and morphological abnormalities. In addition, their study showed that these effects were associated with oxidative stress, inflammatory responses, and disruption of endocrine-related pathways. These results support the hypothesis that glyphosate toxicity involves multiple interconnected mechanisms, contributing to both morphological and physiological alterations during early development.

It was also possible to observe an increase in the body size of embryos exposed to herbicides. Generalized edema is a common manifestation of inflammatory responses to toxic agents in aquatic organisms [42]. In this case, larvae exposed to M1 showed a significant increase in generalized edema compared to the control group, suggesting a more pronounced inflammatory response. This response may contribute to the activation of the antioxidant system, as inflammation can stimulate the production of endogenous antioxidants to counteract the effects of reactive oxygen species (ROS) [7,40].

Considering this, we observed that embryos exposed to both pesticides show a peak of edema for G1 and G2 at 48 h and for D1, D2, and M1 at 72 h. Therefore, exposure to herbicides and their mixtures can lead to edema in zebrafish embryos, with the effect depending on the concentration and composition of the herbicides. Furthermore, it is notable that exposure time played an important role in the formation and decrease in edema since after the maximum incidence there was a reduction in the number of fish with the effect. The process of normalization of the edema effect after the peak suggests that the embryos may have developed some form of tolerance or adaptability to the herbicides over time [43].

After 96 h of exposure, we noticed a significant increase in the occurrence of yolk sac edema in the M1 group compared to the isolated groups (D1, D2, G1, G2) and in relation to the M2 group. In addition, the significant difference between the mixture groups (M1 and M2) and the control group stands out when we consider the sum of the teratogenic effects observed. These results show that the combination of relatively low concentrations of DIC and GLY can intensify the induction of yolk sac edema. This suggests potential interaction effects (additive or synergistic), although this was not formally tested, since the mixture had a more marked teratogenic impact on zebrafish embryos than the isolated herbicides [44]. It is important to note that sublethal effects were more pronounced at lower concentrations, whereas higher concentrations primarily resulted in increased lethality. Consequently, the higher frequency of teratogenic effects observed in the M1 group compared to M2 does not necessarily indicate reduced toxicity at higher concentrations. Instead, these effects may have been masked by the increased mortality observed in M2, limiting the detection of sublethal outcomes.

In addition, up to 2% of the oxygen obtained by a cell can be converted into reactive oxygen species (ROS), which is generated mainly in the mitochondria during aerobic respiration. These molecules are important in signaling processes and cell growth, as well as being essential for the immune response [40]. However, if the production of ROS in the cell exceeds its antioxidant activity, resulting in an accumulation of these compounds, there will be a loss of homeostasis leading to oxidative stress [7,45]. Many ROS are highly reactive with biological macromolecules (DNA, proteins, and lipids), oxidizing them and consequently causing mutations, carcinogenicity, apoptosis, necrosis, and hereditary diseases [46].

To mitigate the harmful effects of the high concentration of ROS, cells have an antioxidant system, which is made up of enzymatic molecules, such as SOD and GPx, and non-enzymatic molecules, such as glutathione [7]. The first ROS formed is the superoxide radical (O_2_^•−^), which is formed when molecular oxygen (O_2_^•−^) receives an electron. The SOD enzyme is responsible for catalyzing O_2_^•−^, dismutating it into H_2_O_2_ and O_2_. Since H_2_O_2_ can generate highly reactive species such as the hydroxyl radical (^•^OH), through the Fenton reaction, it is converted into H_2_O and O_2_ by the GPx enzyme [7,46].

In this study, we obtained a significant reduction in SOD activity after exposure to the herbicide DIC at a higher concentration (D2), which suggests a reduction in the organism’s ability to neutralize the free radical O_2_^•−^, consequently resulting in oxidative stress. In addition, the increase in SOD activity after exposure to GLY in groups G1 and G2 indicates that GLY increases the production of ROS in the body, something already observed in another review article on zebrafish and the herbicide [47], which induced an antioxidant response. This increase in SOD activity may be an attempt by the body to combat the oxidative stress caused by GLY, which was also reported in another study that evaluated the same parameter in zebrafish larvae [48]. Furthermore, the increase in glutathione peroxidase (GPx) activity for the G2 group also suggests that the herbicide may disrupt the body’s antioxidant defense system.

AChE activity is often used as a biological indicator to assess the neurotoxicity of substances. An increase in AChE activity may indicate an adaptive response by the organism to substances that affect neurotransmission [49], which may be a sign that the nervous system is under stress. Additionally, increased expression or activity of AChE is a common feature in apoptotic cells [50,51]. Isolated DIC, both D1 and D2, resulted in a significant increase in AChE activity compared to the control. M2 also showed different effects in relation to control. The increase in AChE activity after exposure to the isolated herbicide and in mixture suggests that the action on the nervous system may be due to DIC, regardless of the presence of GLY.

Exposure to M2 shows no significant difference in edema but affects AChE activity and results in greater lethality. This suggests that, at these concentrations and in a mixture, pesticides may have direct effects on the nervous system and may be more toxic, since lethality is a more serious outcome than edema. Therefore, even though M1 seems to have more effects on the inflammatory response, the severity of the effects caused by M2, such as lethality and neurological problems, shows that this mixture may be more harmful to the embryos. However, it is important to note that the inflammatory process can also trigger other pathways, including those that increase cell death [40] and brain damage [52].

This study provides a solid foundation on the effects of dicamba and glyphosate on zebrafish, demonstrating that these herbicides, even at concentrations considered safe, induce irreversible cellular damage, as well as alterations in important biological parameters such as mortality, teratogenesis, and antioxidant and neurological changes. However, there are still several aspects that warrant further exploration. A more detailed investigation into the molecular mechanisms underlying these effects, such as oxidative stress and inflammation processes, could help clarify how these compounds affect organisms at the cellular and genetic levels. Furthermore, evaluating the combined exposure to DIC and GLY, as it occurs in real environmental scenarios, is particularly relevant. Although this study did not specifically address mechanistic interactions, assessing co-exposure at different concentrations and formulations can provide valuable information about the overall toxicity profile of these herbicides. The work presented here serves as a valuable starting point for such investigations, with the potential to offer important insights for herbicide regulation and their effects in aquatic organisms.

## 5. Conclusions

This study observed that exposure to the herbicides dicamba (DIC) and glyphosate (GLY) during the embryonic period induces significant alterations in zebrafish embryos. These herbicides caused different effects on zebrafish embryos and larvae, even at low concentrations, including those established as safe in drinking water [26]. The lethality, teratogenic effects, edema, and biochemical alterations showed the complexity of the impacts of these herbicides. While GLY showed greater lethality, DIC caused pronounced effects such as edema and malabsorption of nutrients. The effects of the mixture of the herbicides were not consistent across concentrations and endpoints. It was observed that when exposed to lower concentration (M1) there was an increase in teratogenic effects observed, whereas when exposed to M2 the effects were similar to or lower than individual exposures, possibly due to increased lethality masking sublethal outcomes. In addition, biochemical markers suggest the involvement of oxidative stress and neurotoxicity pathways.

Finally, our study advances current knowledge on the herbicide DIC and its effects on zebrafish embryos, particularly in combination with GLY. Given the limited number of studies addressing DIC toxicity, our findings provide novel and important insights. Although zebrafish represent a valuable model for developmental toxicity, caution is warranted when extrapolating these results, and further investigations using complementary models are necessary. Accordingly, future research should incorporate molecular approaches to examine the expression of genes involved in inflammation, apoptosis, and antioxidant defense pathways.

## Figures and Tables

**Table 1:** Teratogenic effects observed in zebrafish embryos after 96 h of exposure to the herbicides DIC and GLY, isolated and in mixture.

Effects (96 h)	C	D1	D2	G1	G2	M1	M2
Eye malformation	0	1	1	4	0	3	2
Tail and spine malformation	1	3	6	5	1	3	1
Cardiac edema	0	1	2	2	0	2	2
Dwarfism	0	1	2	1	1	1	2
Yolk Malabsorption	1	15 *	11	3	1	2	26 *

Values represent the number of occurrences of each alteration. Multiple alterations may occur in a single embryo; therefore, the total number of events may exceed the number of individuals per group. C: Control; dicamba 1 (D1): 18 mg/L; dicamba 2 (D2): 72 mg/L; glyphosate 1 (G1): 6 mg/L; glyphosate 2 (G2): 22 mg/L; mixture 1 (M1): D1 + G1; mixture 2 (M2): D2 + G2. * indicates results significantly different from the negative control (*p* < 0.05), based on one-way ANOVA followed by Tukey’s multiple comparison test (*n* = 60 per group). Differences in survival between groups should be considered when comparing absolute values.

## Data Availability

The raw data supporting the conclusions of this article will be made available by the authors on request.
